# Tranexamic acid inhibits group A streptococci–mediated fibrinolysis *in vitro* and modulates host immune cells *in vivo*

**DOI:** 10.3389/fimmu.2025.1698191

**Published:** 2025-11-14

**Authors:** Maresa Possehl, Praveen Vasudevan, Sonja Schenk, Claudia Maletzki, Robert David, Bernd Kreikemeyer, Sonja Oehmcke-Hecht

**Affiliations:** 1 Institute of Medical Microbiology, Virology and Hygiene, Rostock University Medical Center, Rostock, Germany; 2 Rudolf-Zenker-Institute for Experimental Surgery, Rostock University Medical Center, Rostock, Germany; 3 Department of Medicine, Clinic III-Hematology, Oncology, Palliative Medicine, Rostock University Medical Center, Rostock, Germany; 4 Department of Cardiac Surgery, Rostock University Medical Center, Rostock, Germany; 5 Faculty of Interdisciplinary Research, Department Life, Light & Matter, University Rostock, Rostock, Germany

**Keywords:** txa, fibrinolysis, group a streptococcus, pneumosepsis, innate immune cells

## Abstract

Group A Streptococcus (GAS) exploits the host fibrinolytic system by activating plasminogen via streptokinase, promoting clot degradation, tissue invasion, and immune evasion. Tranexamic acid (TXA), a clinically used antifibrinolytic agent, inhibits fibrinolysis, but its impact on GAS virulence and host immune responses remains incompletely understood. We investigated whether clinically relevant concentrations of TXA or ϵ-aminocaproic acid (AHA) inhibit GAS-induced fibrinolysis, affect bacterial survival in blood, and modulate host immune responses. *In vitro* plasma clot lysis assays, D-dimer quantification, and bacterial escape experiments were used to assess fibrinolytic activity. Western blots and substrate assays evaluated plasminogen and fibrinogen binding and plasmin activity. Bacterial survival and immune phenotypes were analyzed in human blood, and *in vivo* responses were assessed in a murine intranasal infection model. TXA at therapeutic concentrations (10–50 µg/ml) blocked streptokinase- and GAS-induced fibrinolysis, reduced D-dimer release, and prevented bacterial escape from clots *in vitro*. It impaired GAS survival in whole human blood without affecting growth in plasma or culture medium, suggesting a host-mediated effect. TXA affected plasminogen interaction with the bacterial surface and reduced fibrinogen degradation, suggesting interference in GAS-driven fibrinolysis. In infected blood, TXA partially restored CD169 and CD66b expression, consistent with preserved monocyte and neutrophil activation. *In vivo*, TXA lowered lung IL1β and shifted cardiac macrophage polarization toward more M1 and fewer M2 cells. These findings indicate that TXA not only inhibits GAS-induced fibrinolysis but also enhances innate immune responses, exerting both antifibrinolytic and immunomodulatory effects during infection.

## Introduction

Group A Streptococcus (GAS) is a major human pathogen responsible for a range of diseases, from superficial infections to invasive syndromes such as necrotizing fasciitis and streptococcal toxic shock syndrome. A hallmark of GAS virulence is its ability to hijack the host fibrinolytic system by secreting streptokinase, which activates plasminogen into plasmin. Surface-bound plasmin promotes degradation of fibrin clots and extracellular matrix components, thereby facilitating bacterial invasion, dissemination, and immune evasion (1).

Antifibrinolytic agents such as tranexamic acid (TXA) and ϵ-aminocaproic acid (AHA) are widely used in clinical settings to reduce bleeding (2). These lysine analogs competitively block plasminogen binding to fibrin and other substrates by targeting lysine-binding sites (LBS) in the Kringle domains of plasminogen (3). Although their hemostatic efficacy is well documented, less is known about their potential to interfere with bacterial exploitation of host fibrinolysis, particularly at concentrations routinely used in patients.

Recent studies suggest that TXA may also modulate bacterial survival and immune responses, but these effects are incompletely understood and often observed at supratherapeutic concentrations (4). Whether therapeutically relevant doses of TXA can affect GAS virulence mechanisms—such as fibrinolysis or immune evasion—remains to be elucidated.

In this study, we examined whether TXA and AHA at clinically relevant concentrations interfere with GAS-induced fibrinolysis using *in vitro* clot degradation assays, D-dimer release, and bacterial escape models. We further explored the effect of TXA on GAS survival in human blood, and assessed how TXA modulates immune cell activation and host–pathogen interactions, both *ex vivo* and in a murine pneumosepsis model. Our findings reveal that TXA not only impairs fibrinolysis and bacterial escape but also enhances immune-mediated clearance of GAS in blood, suggesting a dual benefit of TXA in invasive bacterial infections—mechanical inhibition of fibrinolysis and restoration of innate immune function.

## Material and methods

### Bacterial strains and culture conditions

The *S. pyogenes* strain AP1 (40/58) has been described (5).

#### Human plasma

If not otherwise indicated plasma from 3 healthy individuals was obtained from the blood bank at Rostock University Hospital, Rostock, Germany, kept frozen at 80 °C and pooled before use.

#### Clot lysis time

A clot was generated in pooled human plasma by addition of PT reagent (Haemochrom Diagnostica), containing TXA (Sigma-Aldrich) or AHA (Sigma-Aldrich) at 10 or 20 µg/ml or PBS as control. The clot was incubated for 2 min at 37 °C before streptokinase (10 Units, Sigma-Aldrich) was added. In some experiments a logarithmic GAS suspension (2x10^9^ CFU/ml) and AHA or TXA at indicated concentrations were added before clot formation was induced by addition of thrombin. Time until clot lysis was determined in a coagulometer (6).

#### D-dimer ELISA

Samples were generated by mixing logarithmic GAS bacteria (2x10^8^ CFU/ml) 1:1 with pooled human plasma and AHA or TXA at indicated concentrations. Clot formation was induced by addition of thrombin (5 Units) and incubated for 5 min at 37 °C. The clot was covered with PBS (containing 1% plasma) and incubated at 37 °C. Samples were taken from the supernatant at indicated time points and stored at -20 °C. The D-dimer ELISA (Technoclone) of the samples was performed according to the manufacturer’s instructions.

#### Plasma clot escape experiments

Pooled plasma was mixed with logarithmic GAS bacteria (10^5^ CFU/ml) and TXA (10 µg/ml), and a stable clot was induced by addition of thrombin (10 Unit). The clot was overlaid with PBS, containing 1% plasma. After 2 and 4 hours (h), the bacterial loads in the supernatant and homogenized clots were determined by plating.

#### Bacterial survival in human plasma and blood

An overnight GAS culture was set to 2x10^9^ CFU/ml in PBS and mixed with human plasma (pooled from 4 healthy donors, n=4 experiments) or blood from 9 different healthy donors, and TXA at indicated concentrations. In the control, buffer was used instead of TXA. After a four-hour incubation period at 37 °C, the surviving bacteria were determined by plating and the multiplication factor was calculated from the number of the initially added bacteria and the number of the surviving bacteria.

#### Immune phenotyping of human blood

Blood from 5 healthy donors was incubated with GAS (5x10^3^ CFU/ml) in the presence or absence of TXA (10 and 20 µg/ml). After 4 hours of incubation at 37 °C, the blood samples were incubated with the indicated antibodies (CD3, CD14, CD16, CD19, CD56, CD66b, CD169) and measured on the *FACSVerse* flow cytometer as described before (7). Data acquisition was done using FACSSuite version 1.0.6.5230 software (Becton Dickinson), followed by data analysis using FlowJo version 10.10.0.

#### Plasma protein adsorption and western blot analysis

For sampling, GAS overnight cultures were set to 1×10^10^ CFU/ml in PBS, mixed with an equal amount of pooled human plasma and TXA at indicated concentrations, and incubated at 37 °C for 30 min. Incubation of plasma with PBS, and bacteria with PBS served as controls. After centrifugation, bacterial pellets were washed 3 times (6800xg, 5 min) with PBS, and the pellet was resuspended in glycine (0.1 M), and incubated at room temperature for another 10 min. The pH value of supernatants from subsequent centrifugation (12000xg, 5 min) was neutralized by the addition of Tris-HCl (1 M, pH = 8.4), and the suspensions were mixed with SDS sample buffer (5x). Sampling was performed on 3 different days. SDS-PAGE was performed as described earlier (8). Following SDS-PAGE, separated proteins from the eluates were transferred onto nitrocellulose membranes. Western blot analyses were performed with anti-human fibrinogen (alpha chain, Santa Cruz), or anti-plasminogen (Santa Cruz). Blots were incubated with secondary fluorophore-labeled antibodies (LI-COR) and imaged using an Odyssey Imager (LI-COR).

## Ethics approval statement

The protocol for the collection of human blood was approved by the ethics committee at the medical faculty of the University of Rostock (ethics committee vote: A 2014-0131). The experiments were conducted in accordance with the ICH-GCP guidelines. Written informed consent was obtained from all donors prior to donation, and all samples were anonymized before experimental use.

## Animal experiments

The experiments were performed with humanized plasminogen transgenic mice (*AlbPLG1*) (9) and animals were infected as described before (7). Briefly, ten-to-twelve-week-old female *AlbPLG1* mice with a weight of 20–22 g were infected intranasally with 20 µl of 1.5 x 10^8^ CFU bacteria (strain AP1), diluted in PBS. Four, 24 and 30 h after infection n=7 animals were treated with TXA (200 µg/mouse in 20 µl) intranasally. The control group (n=6) received saline (20 µl) at the same time points. 48 h after infection animals were euthanized and blood and organs collected. The protocol was approved by the Committee on the Ethics of Animal Experiments the Landesveterinär- und Lebensmitteluntersuchungsamt Rostock (Permit Number: 7221.3-1-056/17).

### Lung homogenates from mice

Left lung was harvested and prepared as described before (7).

### D-dimers in lung

The quantitative determination of D-dimers in lung homogenates was performed as described before (7).

### Clotting times

All clotting times in mouse plasma were measured as described previously (6, 10).

### Peripheral blood smear analysis

Slides were stained by Coomassie`s staining with GIEMSA kit (MORPHISTO). One hundred white blood cells were counted and categorized into granulocytes, lymphocytes and monocytes.

### Cytokine ELISA from lung homogenates

The concentrations of IL6, TNFα, IL1β, IL8, IFNγ and IL10 in lung homogenates were determined by ELISA according to the manufacturer’s protocol (R&D Systems).

### Immune phenotyping of heart tissue

The heart was used for immune phenotyping as described before (7).

### Statistics

All values are reported as mean ± SD from at least n=3 experiments, if not otherwise stated. Data were tested for normality in GraphPad Prism using the Shapiro–Wilk, D’Agostino & Pearson, Anderson–Darling, and Kolmogorov–Smirnov tests. Normality was assumed if at least one of these tests indicated a normal distribution. Differences between controls and treated samples were determined by using the unpaired or paired t-test. If normality failed, the non-parametric Mann-Whitney U-Test was applied. In case of multiple comparisons one- or two-way ANOVA on ranks was applied, with Dunnett`s posttest (to compare with the control) or the Sidak`s posttest (to compare preselected pairs of columns). Statistical evaluation was performed using GraphPad PRISM software, version 10.5.0 (GraphPad Software, San Diego, CA, USA). The criterion for significance was taken to be p < 0.05.

## Results

### Therapeutic concentrations of TXA and AHA inhibit clot lysis induced by streptokinase or GAS bacteria

**Figure 1:**
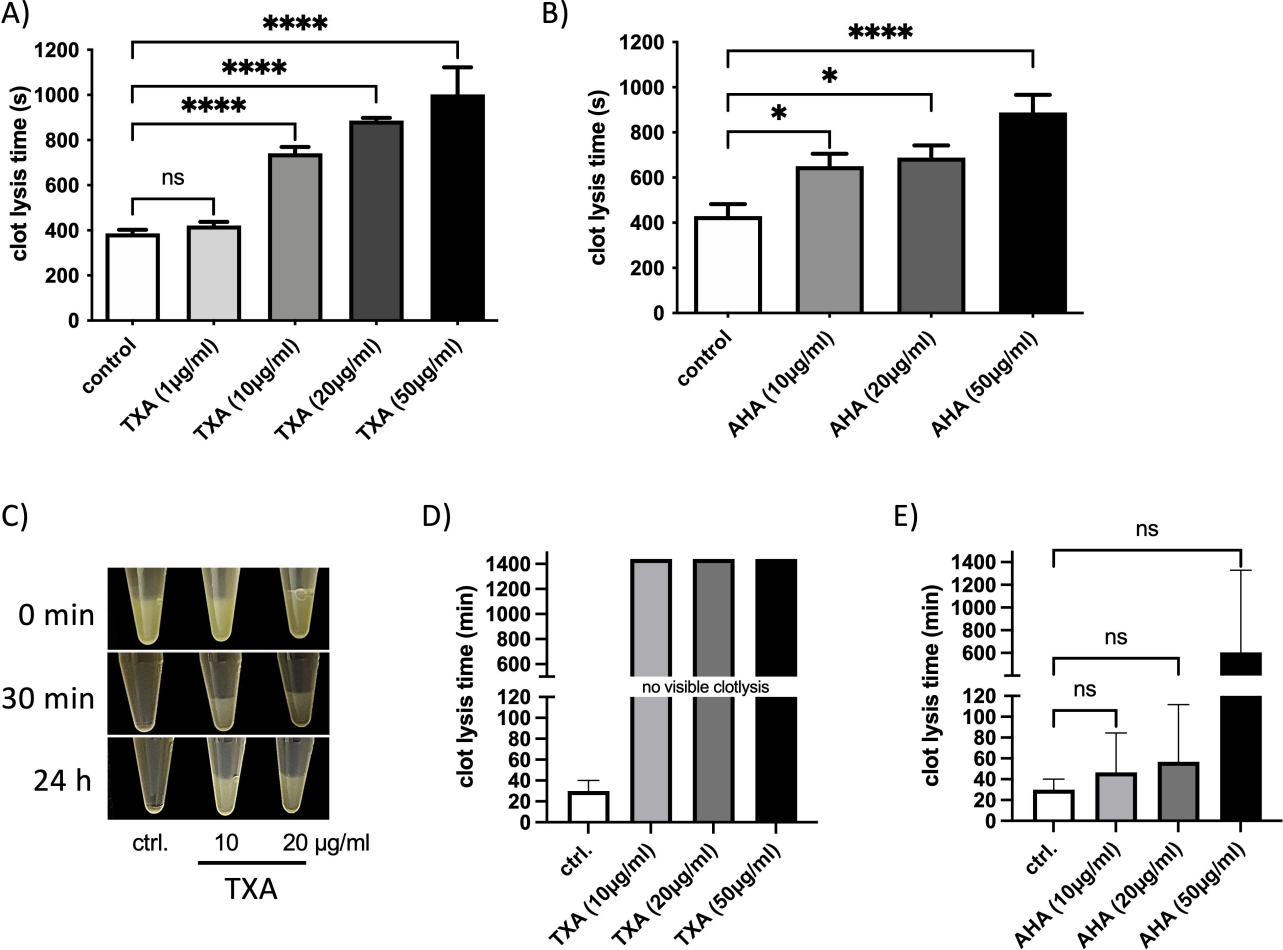
Clot lysis – induced by streptokinase or GAS – is inhibited in the presence of therapeutic doses of TXA and AHA. Pooled plasma with buffer (control) or TXA **(A)** AHA **(B)** at concentrations of 1, 10, 20, and 50 µg/ml, respectively was placed in a coagulometer. Clot formation was initiated by the addition of thromboplastin. Following an additional incubation period of 2 minutes, streptokinase was administered to the samples, and the time to clot dissolution was recorded. Data are presented as mean values ± standard deviations (n = 7 experiments for TXA and n = 8 experiments for AHA). Statistical significance was assessed using one-way analysis of variance (ANOVA) followed by Dunnett’s *post hoc* test. *p < 0.0107, **p < 0.0099, ***p < 0.0002, ****p < 0.0001. **(C-E)** A plasma clot was generated by incubating pooled plasma with a logarithmic GAS suspension (2x10^9^ CFU/ml), thrombin (1 Unit), TXA or AHA at 10 or 20 µg/ml or PBS as control (ctrl.). The clot was overlaid with PBS and the time to complete dissolution was documented photographically **(C)**, or measured in a coagulometer **(D, E)**. The data show the mean values and standard deviations of n=3 different experiments. The positive control was chosen as the reference when calculating significance in the one-way ANOVA with Dunnett’s test.

TXA concentrations between 10 and 15 µg/ml result in substantial inhibition of fibrinolysis in patients (11), we therefore first investigated whether TXA or AHA at these concentrations inhibit streptokinase-induced clot lysis. For this *in vitro* plasma clot lysis assays were performed where streptokinase was incubated with a plasma clot and the time until clot lysis was measured. Clot lysis time was significantly prolonged in the presence of TXA (from 10 µg/ml, **Figure 1A**) or AHA (from 10 µg/ml, **Figure 1B**).

Next, we assessed whether TXA or AHA could inhibit clot degradation mediated by GAS bacteria. Plasma clots incubated with logarithmic GAS bacteria underwent complete lysis within 30 minutes (**Figures 1C–E**). However, when TXA was present at concentrations of 10, 20 or 50 µg/ml, the clots remained intact and visible, even after 24 hours of incubation (**Figures 1C, D**), indicating potent inhibition of GAS-mediated fibrinolysis.

In contrast, the presence of AHA resulted in variable lysis times, and statistical significance could not be established compared to the untreated control (**Figure 1E**). Overall, these results show that TXA is more effective than AHA at inhibiting fibrinolysis induced by either streptokinase or GAS under the tested conditions.

### TXA and AHA reduce D-dimer levels and inhibit bacterial escape from plasma clots

**Figure 2:**
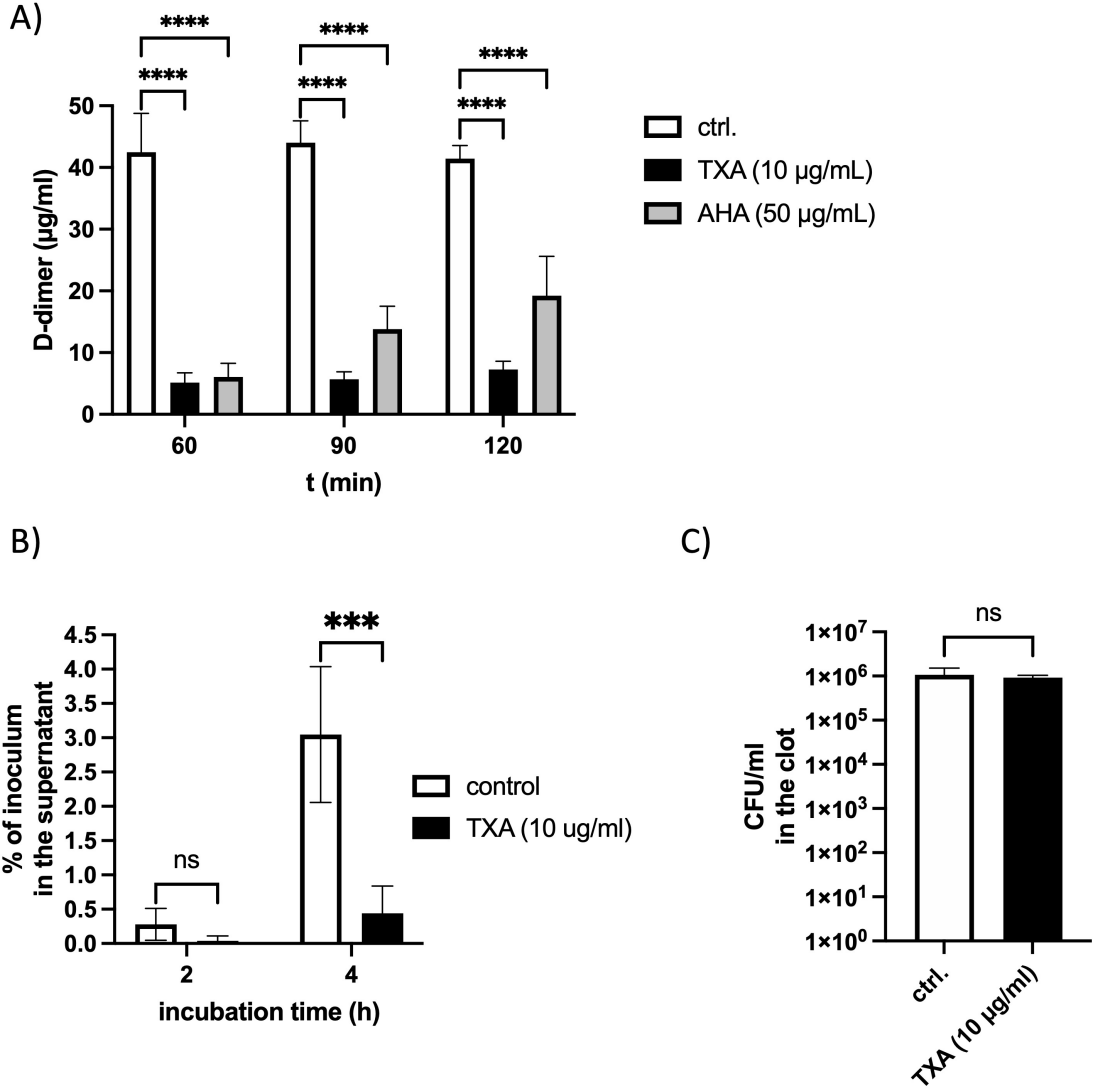
D-dimer release and bacterial escape from plasma clots are inhibited in the presence of TXA or AHA **(A)** Logarithmic GAS (2x10^8^ CFU/ml) were mixed with pooled plasma, TXA (10 µg/ml), AHA (50 µg/ml) or PBS as control (ctrl.), and thrombin (5 Units) was used to form a stable clot. The plasma clot was overlaid with PBS and D-dimer concentration in the supernatant was measured after different time points, using an ELISA. The data show the mean values and standard deviations of n=3 different experiments. Two-way ANOVA with Dunnett’s test comparing to the control. ****p<0.0001. **(B)** Pooled plasma was mixed with 10^5^ CFU/ml GAS bacteria and TXA (10 µg/ml) and a stable clot was induced by addition of thrombin (1 Unit), and overlaid with PBS, containing 1% plasma. After 2 and 4 hours (h), the bacterial loads in the supernatant **(B)** and homogenized clots **(C)** were determined by plating. The data were normalized to the number of inoculated bacteria. n=3 different experiments. Two-way ANOVA with Sidaks posttest. ***p=0.0008 **(B)** or unpaired t-test **(C)**.

Since fibrinolysis leads to the release of D-dimers from cross-linked fibrin, we quantified D-dimer levels in supernatants of plasma clots incubated with GAS bacteria in the presence or absence of TXA (10 µg/ml) or AHA (50 µg/ml). After 60 minutes, D-dimers were detected in all samples; however, their concentrations were significantly reduced in the presence of either TXA or AHA (**Figure 2A**). Notably, D-dimer levels did not increase over 120 minutes in TXA-treated samples, indicating potent inhibition of GAS-induced fibrinolysis.

To determine whether TXA also affects the ability of GAS to escape from clots, human plasma containing GAS and TXA (10 µg/ml TXA) was clotted with thrombin. Clots were overlaid with 1% plasma and incubated at 37 °C. Viable bacterial counts were assessed in the supernatant after 2 and 4 hours. At 2 hours, very few bacteria were detected in the supernatant. After 4 hours, supernatants from TXA-treated samples contained significantly fewer colony-forming units (CFUs) compared to untreated controls (**Figure 2B**), indicating that TXA limits bacterial escape. Importantly, bacterial counts within the clots were unchanged (**Figure 2C**), demonstrating that TXA does not exert a bactericidal effect under these conditions.

### Therapeutic concentrations of TXA reduces GAS survival in human blood

Previous studies have shown that high concentrations of TXA exhibit antibacterial activity against pathogens such as *Staphylococcus aureus* and *Cutibacterium acnes* (12). To assess whether TXA affects the growth of GAS, we examined bacterial proliferation in laboratory medium, human plasma, and whole blood.

**Figure 3:**
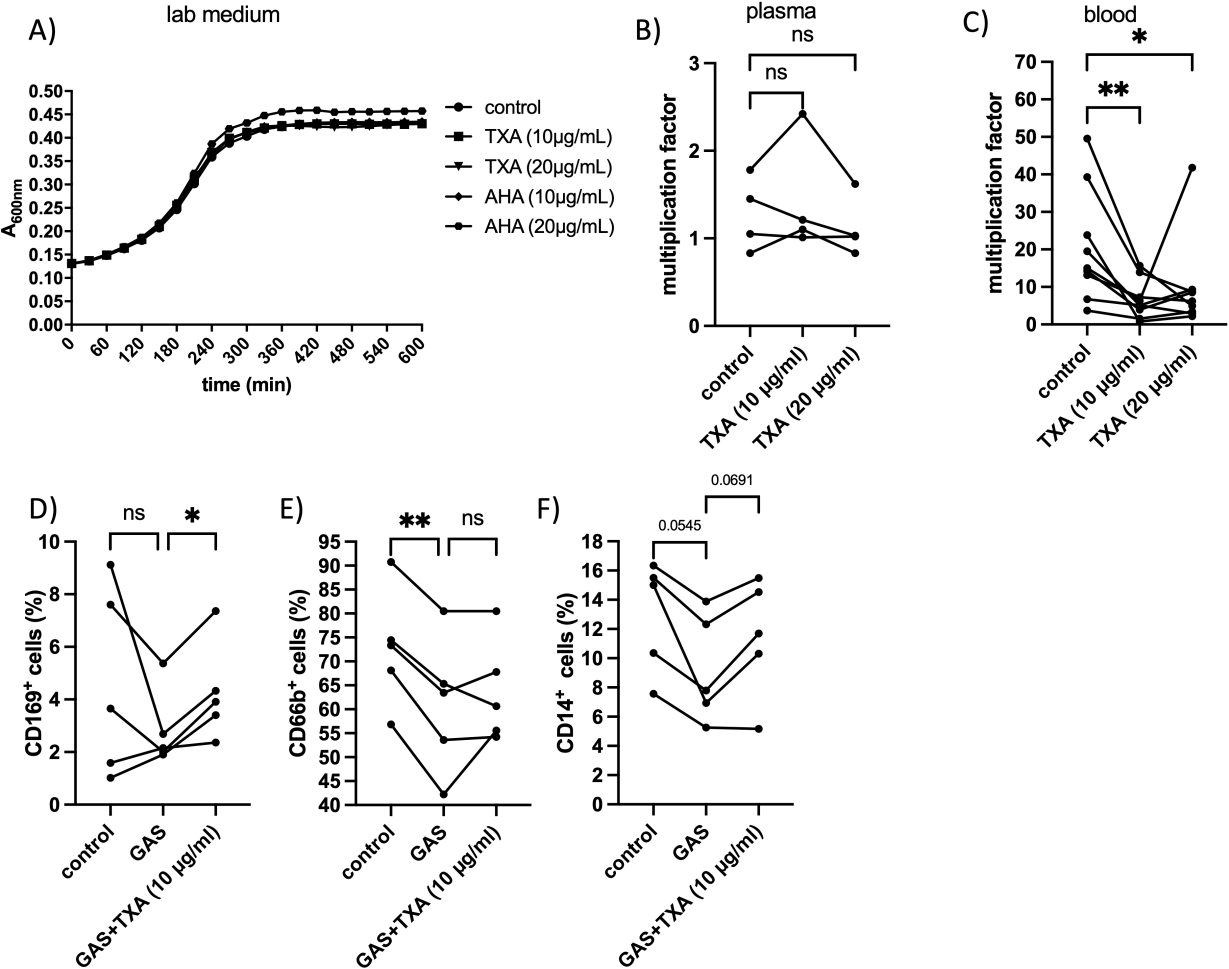
GAS growth and multiplication factor and changes in immune cell markers after treatment with TXA and/or AHA. **(A)** Bacteria were incubated in lab medium with TXA and AHA at indicated concentrations, or PBS as control. The increase in optical density at 600 nm was measured photometrically. **(B)** An overnight GAS culture (2x10^9^ CFU/ml) was mixed with pooled human plasma (derived from 4 healthy donors) and TXA at indicated concentrations. In the control, buffer was used instead of TXA. After a four-hour incubation period at 37°C, the surviving bacteria were determined by plating and the multiplication factor was calculated from the number of the initially added bacteria and the number of the surviving bacteria. n=4 different experiments. Significance was tested using the Friedman test followed by the Dunn test. **(C)** Human whole blood was incubated with GAS in the early exponential growth phase and TXA at indicated concentrations or buffer for the control. After a four-hour incubation period at 37°C, the surviving bacteria were determined by plating and the multiplication factor was calculated from the number of the initial added bacterial and the number of the surviving bacteria. Blood from 9 healthy donors was used. Significance was determined using the Friedman test followed by the Dunn test *p=0.0190, **p = 0.0044. **(D-F)** Citrated blood was incubated with GAS in the presence or absence of TXA (10 and 20 µg/ml). After 4 hours of incubation at 37°C, the blood samples were incubated with anti CD169 **(D)**, anti CD66b **(E)** or anti CD14 **(F)** and measured on the FACSVerse (see material methods). Blood from five healthy donors was used. Significance was determined using the RM one-way ANOVA for paired samples followed by Dunnett’s test; **p < 0.0055. In the experiment with the CD169 antibody the Friedman test followed by Dunn’s test was used.

TXA at 10 – 20 µg/ml had no detectable effect on GAS growth in standard laboratory medium (**Figure 3A**) or pooled human plasma (**Figure 3B**). However, in whole blood, TXA at these concentrations significantly reduced GAS proliferation (**Figure 3C**). These findings suggest that TXA does not directly inhibit GAS growth, but rather exerts an indirect effect mediated by immune cells.

To investigate this further, we analyzed the expression of immune cell surface markers in blood incubated with GAS in the presence or absence of TXA. Among the markers examined, only CD169 and CD66b showed significant alterations (**Figures 3D, E**). The frequency of CD169 positive cells significantly increased following incubation with GAS and TXA (10 µg/ml), compared to GAS-only conditions (**Figure 3D**), suggesting an enhanced monocyte/macrophage response. CD66b-positive cells (granulocytes) were significantly reduced following GAS infection compared to buffer-treated controls, and TXA treatment led to partial restoration of CD66b levels in 2 out of 5 donors (**Figure 3E**).

CD14-positive cells (monocytes) decreased in all donors upon GAS exposure, and TXA treatment partially restored CD14 levels in 4 of 5 donors. However, this trend did not reach statistical significance (**Figure 3F**).

Together, these findings suggest that TXA modulates immune responses in whole blood, possibly enhancing the ability of host cells to control GAS infection.

### TXA modifies plasminogen interaction with the GAS surface and reduces fibrinogen degradation without affecting plasmin activity.

**Figure 4:**
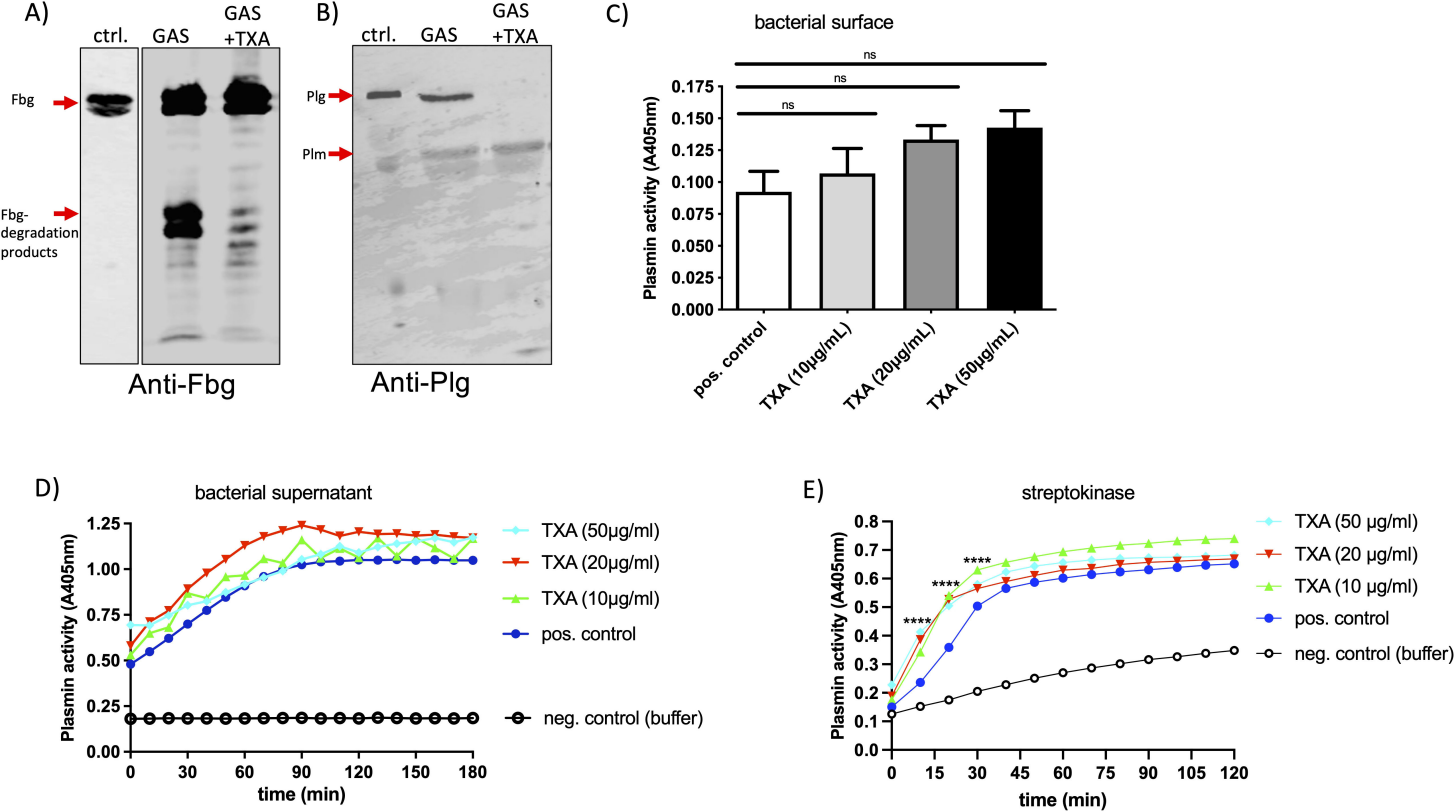
TXA reduces fibrinogen degradation and plasminogen binding to the GAS surface, without inhibiting plasmin activity. Bacteria (1x10^10^ CFU/ml) were incubated in pooled plasma in the presence of 10 µg/ml TXA (GAS+TXA). After incubation bacteria were washed and the adsorbed proteins were eluted from the surface. Western Blot analysis of the eluates was performed with an antibody against fibrinogen [**(A)**, anti-Fbg] or plasminogen [**(B)**, anti-Plg]. As control (ctrl.) plasma with buffer was used. **(C)** GAS-bacteria (2x10^9^ CFU/ml) were incubated in pooled plasma (1:1) in the presence of indicated TXA concentrations. After incubation bacteria were washed three times with Tris-buffer and plasmin activity was determined using the substrate S-2403, specific for plasmin. Significance was tested by One-way ANOVA and Dunnett’s multiple comparisons test. **(D, E)** Bacterial supernatant **(D)** or streptokinase **(E)** was mixed with pooled plasma and indicated TXA concentrations, S-2403 was added and absorption was measured. Significance was tested by two-way ANOVA and Tukeys multiple comparisons test, ****p<0.0001 at indicated time points.

As TXA interferes with binding of plasminogen or plasmin to the lysin residues in fibrin, we investigated whether it might affect binding of plasminogen and fibrinogen to the GAS surface. GAS bacteria were incubated in plasma in the presence or absence of TXA, washed and the adsorbed proteins were eluted from the surface. Eluates were investigated by Western blot analysis for their containment of plasminogen or fibrinogen. Fibrinogen in plasma showed two prominent signals between 70 and 100 kDa (**Figure 4A**, ctrl.), whereby fibrinogen - eluted from the bacterial surface – showed two additional signals between 40–55 kDa (**Figure 4A**, GAS), indicating degradation at the GAS surface. In the presence of 10 µg/ml TXA the fibrinogen degradation products were markedly reduced (**Figure 4A**, GAS+TXA). Plasminogen appeared as a single signal at 90 kDa in the plasma control (**Figure 4B**, ctrl.) and an additional signal at around 60 kDa was detected in the eluates, suggesting that plasminogen is activated to plasmin at the bacterial surface (**Figure 4B**, GAS). However, in the presence of TXA the plasminogen signal disappeared completely and only a faint signal at 60 kDa was detected in the eluate (**Figure 4B**, GAS+TXA). This indicates that either plasminogen no longer binds to the bacterial surface, or the plasminogen (bound to the surface) has been completely converted to plasmin in the presence of TXA.

Next, we assessed plasmin activity at the bacterial surface using a plasmin-specific substrate assay. Neither TXA (**Figure 4C**) nor AHA (not shown) inhibited plasmin enzymatic activity significantly under these conditions, suggesting that TXA’s inhibitory effect on clot lysis is not due to direct inhibition of plasmin activity, but rather results from reduced plasminogen and fibrinogen binding to bacteria. When we tested the GAS supernatant (**Figure 4D**) or streptokinase (**Figure 4E**) a robust plasmin activity was measured, which was not inhibited by the presence of TXA (**Figures 4D, E**) or AHA (not shown). Plasminogen activation - triggered by streptokinase - was even faster and also significantly increased with increasing TXA concentrations (**Figure 4E**).

### TXA enhances streptokinase-induced activation of plasminogen and contact system components

**Figure 5:**
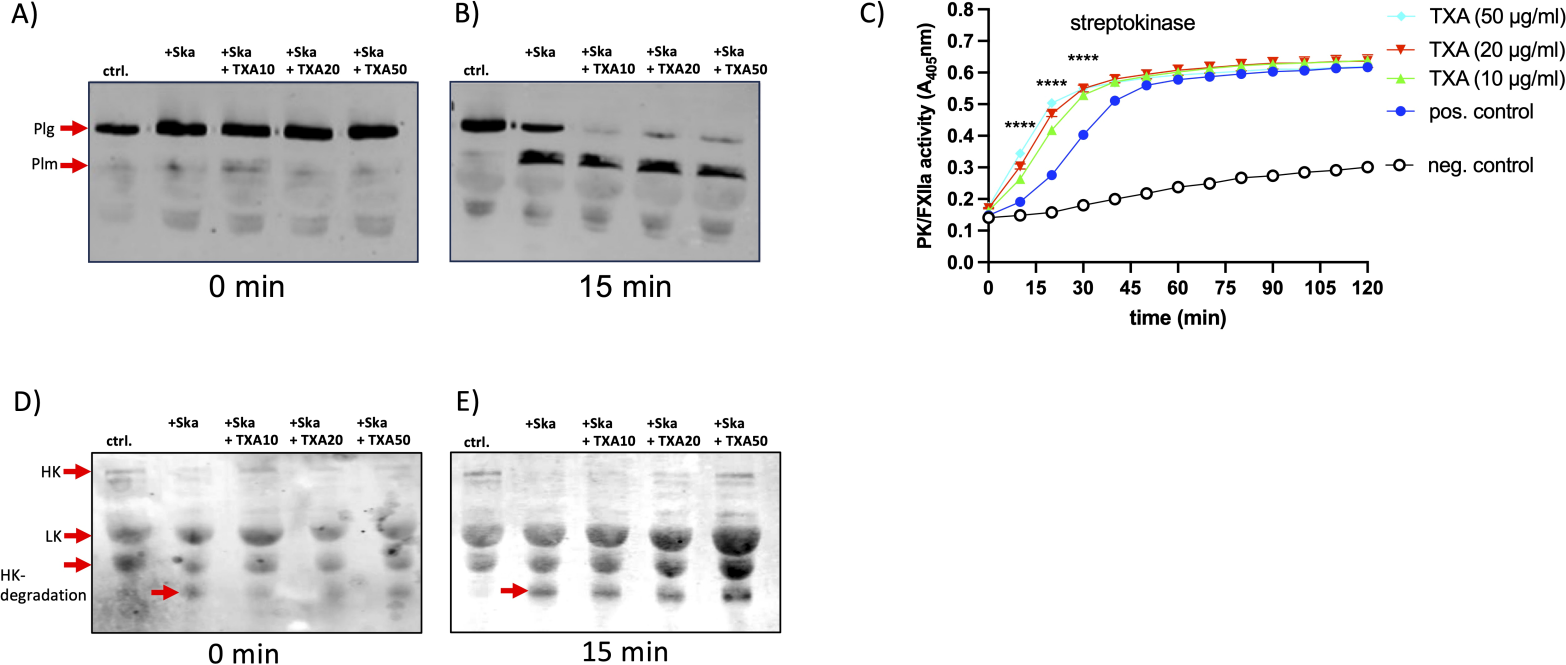
TXA slightly enhanced plasmin- or plasma kallikrein activity, induced by streptokinase Streptokinase (Ska, 10 Units) was incubated in pooled plasma with TXA at 10 (Ska+TXA10), 20 (Ska+TXA20) or 50 µg/ml (Ska+TXA50). PBS instead of streptokinase was used as control (ctrl.). Samples at time point zero and after 15 min were investigated by Western blot with an antibody against plasminogen (plg, **(A, B)** or HK **(D, E)**. **(C)** Streptokinase was mixed with plasma and indicated TXA concentrations, S-2303, specific for PK activity, was added and absorption was measured. Significance was tested by two-way ANOVA and Tukeys multiple comparisons test, ****p<0.0001 at indicated time points.

Increased plasmin activity after activation by urokinase or tPa and in the presence of TXA has been described previously (13), thus, we investigated whether the conversion of plasminogen to plasmin - induced by streptokinase - is affected in the presence of TXA. Streptokinase and increasing concentrations of TXA were incubated in plasma for 15 min and Western Blot analysis, to detect plasminogen, was performed. At time point zero plasminogen appeared as a single signal at 90 kDa in all samples (**Figure 5A**), and after 15 min plasminogen and plasmin are detected in the sample containing streptokinase. In the presence of TXA the signal for plasminogen clearly disappeared, and at the same time plasmin was detected (**Figure 5B**). This suggests that plasminogen is rapidly processed to plasmin by streptokinase, and this process is even faster in the presence of TXA.

We showed previously that GAS triggers contact activation by streptokinase and plasminogen (14), thus we tested plasma kallikrein (PK) activity and degradation of high molecular weight kininogen (HK) in plasma, after incubation with streptokinase and TXA. However, as for plasmin activity, PK activity was not inhibited by increasing concentrations of TXA, on the contrary, activation was significantly faster and increased (**Figure 5C**). We performed Western blot analysis of the plasma samples incubated with streptokinase and TXA, to investigate degradation of high molecular weight kininogen (HK), which is the consequence from PK activation (15). At time point zero and after 15 min HK appeared as a single signal at 120 kDa in the control sample (**Figures 5D, E**, ctrl.). The polyclonal antibody against HK detects also low molecular weight kininogen (LK) at 60 kDa, a shorter splice variant of HK (16). In streptokinase-incubated plasma HK was immediately processed, as the HK signal disappeared completely and HK-degradation products at 45 kDa appeared (**Figure 5D**, +Ska). Incubation with TXA did not prevent HK degradation (**Figure 5E**, Ska+TXA). However, HK remained partially detectable in TXA-treated samples at higher concentrations, suggesting that its processing is incomplete (**Figure 5E**, Ska+TXA50).

### Intranasal TXA treatment does not affect bacterial dissemination, coagulation, or immune cell profiles in a murine intranasal infection model

**Figure 6:**
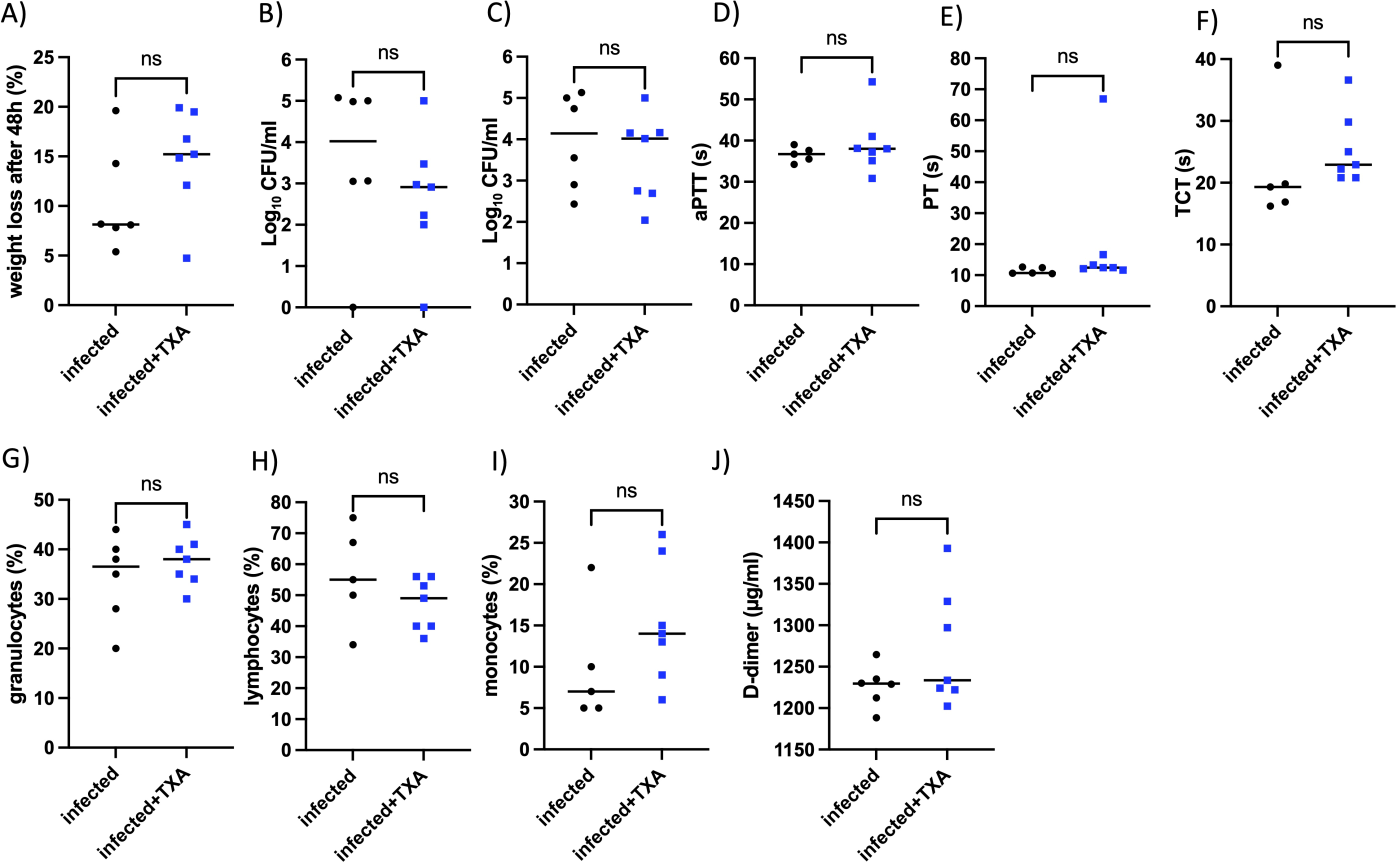
Intranasal TXA treatment does not affect bacterial numbers in lung and spleen, coagulation parameters, or immune cell profiles *in vivo*. *AlbPlg1* mice (n=13) were infected intranasally with 1.5x10^8^ CFU/mouse of *Streptococcus pyogenes* AP1. 4, 24 and 30 h after infection one group (n=7) were treated with TXA intranasally (200 µg/mouse, infected+TXA), and the other group (n=6, infected) recieved saline. 48 h after infection animals were released and blood and organs (lung and spleen) were collected. **(A)** Weight loss after infection. **(B)** CFU determined in right lung. **(C)** CFU determined in spleen. **(D)** aPTT **(E)** PT **(F)** TCT measured in plasma. Granulocytes **(G)**, lymphocytes **(H)**, and monocytes **(I)** were counted in blood smears. **(J)** D-dimers were determined in the homogenized left lung. The statistical differences were tested using t-test or Mann Whitney test.

To evaluate the effect of TXA treatment on the course of GAS infection, we employed a previously established intranasal infection model, using *AlbPLG1* mice (7), which express human plasminogen (9). Mice were infected intranasally with 1.5x10^8^ CFU of GAS. TXA was administered intra-nasally at a dose of 200 µg/mouse 4-, 24-, and 30-hours post-infection. Control animals received an equivalent volume of saline. Overall, mice appeared clinically normal after infection and treatment; however, animals treated with TXA exhibited a more pronounced weight loss after 48 h, compared to controls (**Figure 6A**), however this was not statistically different. At 48 hours post-infection, mice were euthanized and bacterial dissemination was assessed. Bacterial loads in the lung and spleen were comparable between TXA-treated and control groups (**Figures 6B, C**), indicating that TXA did not significantly alter GAS colonization or dissemination at the used concentration.

To assess whether TXA influenced coagulation, we measured standard clotting parameters. No significant differences were observed in activated partial thromboplastin time (aPTT), prothrombin time (PT), or thrombin clotting time (TCT) between the two groups (**Figures 6D–F**), suggesting that TXA, in the concentration applied here, did not markedly alter systemic coagulation.

Peripheral blood smears were analyzed to determine immune cells distributions. No differences were observed in the proportions of granulocytes, lymphocytes, or monocytes between TXA-treated and control mice (**Figures 6G–I**). Additionally, plasma D-dimer levels in the lungs, as marker of fibrinolysis, were not significantly different between the groups (**Figure 6J**).

These results suggest that intranasal administration of TXA, at the given dose and schedule, does not affect GAS dissemination, systemic coagulation, fibrinolytic activity, or peripheral immune cell counts during GAS infection in this murine model.

### TXA treatment modestly reduces inflammation markers in the lungs of infected mice

**Figure 7:**
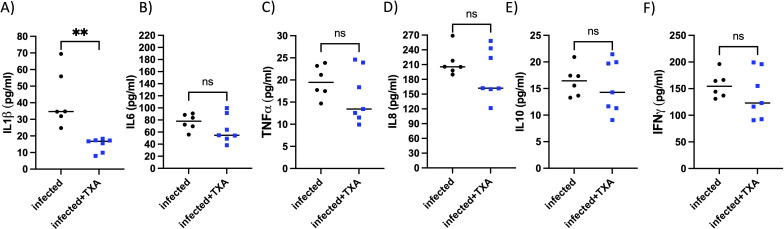
Il1β, but no other tested cytokines, was significantly reduced in the lung of TXA treated mice, infected intranasally with GAS. *AlbPlg1* mice (n=13) were infected intranasally with 1.5x10^8^ CFU/mouse of *Streptococcus pyogenes* AP1. 4, 24 and 30 h after infection one group (n=7) were treated with TXA intranasally (200 µg/mouse, infected+TXA), and the other group (n=6, infected) received saline. 48 h after infection animals were released and the lung homogenates were investigated for **(A)** IL1β, Man-Whitney test, **p=0.0012, **(B)** IL6, **(C)** TNFα, **(D)** IL8, **(E)** IL10 and **(F)** IFNγ by ELISA.

To assess pulmonary inflammation, left lungs were homogenized and cytokine levels were quantified. TXA treatment resulted in a significant reduction of the proinflammatory cytokine IL-1β compared to controls (**Figure 7A**). Levels of other inflammatory markers, including IL-6, TNF-α, IL-8, IL-10, and IFN-γ, were also lower in the TXA-treated group, although these differences did not reach statistical significance (**Figures 7B–F**). These findings suggest that intranasal applied TXA may modestly attenuate lung inflammation during GAS infection.

### TXA treatment selectively modulates cardiac macrophage polarization during systemic GAS infection

In earlier work using the *AlbPLG1* mouse model, we showed that intranasal GAS infection with bacteremia leads to marked changes in cardiac immune cell composition, including increased neutrophils and pro-inflammatory Ly6C^hi monocytes, as well as a shift toward M1-like macrophages and a reduction in anti-inflammatory subsets (7). In the current study, we examined whether TXA treatment influences this cardiac immune landscape.

**Figure 8:**
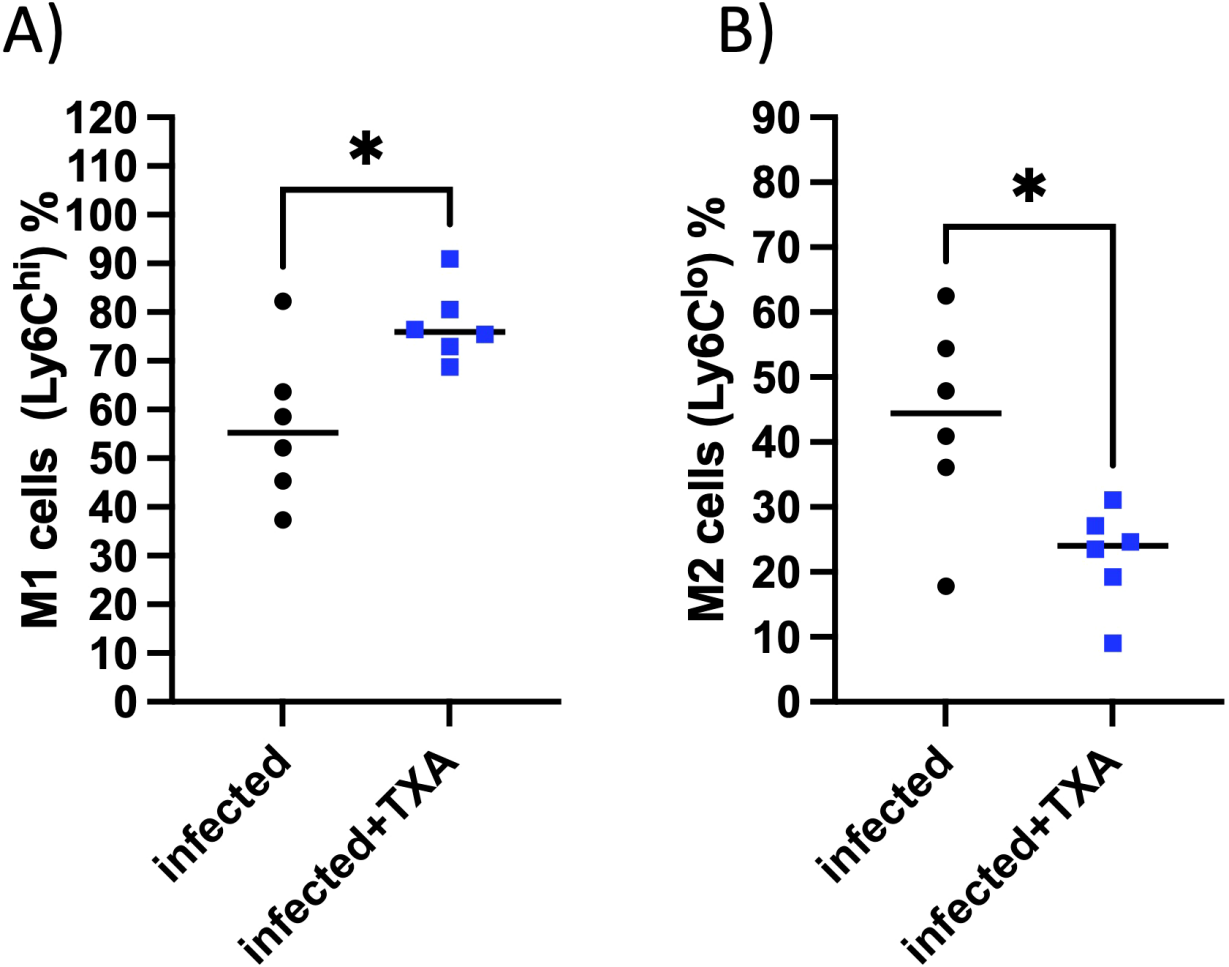
TXA treatment increases M1-like macrophages and reduces M2-like macrophages in the heart during GAS infection. Flow cytometric analysis of cardiac immune cell populations in *AlbPLG1* mice 48 h after intranasal GAS infection and treatment with TXA (infected+TXA, n=6) or vehicle (infected, n=6). **(A)** proinflammatory M1-like macrophages, **(B)** anti-inflammatory M2-like macrophages, unpaired ttest, *p¾ 0.05.

Flow cytometric analysis revealed that intranasal TXA administration during GAS infection did not alter the total number of cardiac CD11b^+^ myeloid cells, neutrophils, or NK cells (not shown). Likewise, the distribution of CCR2^+^ and CCR2^-^ macrophage subsets, and most monocyte populations, remained unchanged between both groups (not shown). However, TXA treatment was associated with a significant increase in M1-like macrophages (Ly6C^hi^, **Figure 8A**), and a decrease in M2-like macrophages (Ly6C^lo^, **Figure 8B**). These changes suggest a selective modulation of macrophage polarization, rather than a broad suppression or recruitment effect.

## Discussion

As GAS use the human fibrinolytic system for dissemination (9) we aimed to investigate whether blocking of fibrinolysis might prevent GAS dissemination and modulate immune responses. Sun et al. (17) already showed that inhibition of *ska* gene expression improves survival after subcutaneous GAS infection in mice. In addition, other substances, namely linoleic and palmitoleic acids, have been found to block streptokinase-mediated plasminogen activation and reduce the severity of invasive GAS infection originating from a subcutaneous site (18). We therefore investigated whether the lysine analogs TXA and AHA, already used clinically to block fibrinolysis, also inhibits GAS-induced fibrinolysis at clinically relevant concentrations. For inhalation applications in humans 250–500 mg/dose are used (19, 20). If applied intravenously TXA concentrations of 10–15 mg/l were sufficient to achieve a substantial level of fibrinolysis inhibition (11). In the present study *in vitro* clot lysis - induced by streptokinase or GAS bacteria – was significantly prolonged with TXA and AHA at 10 mg/l. Moreover, TXA prevented D-dimer release and reduced bacterial escape from plasma clots, indicating that a TXA concentration of 10 mg/l is sufficient to inhibit GAS dissemination.

TXA also appeared to alter plasminogen activation and fibrinogen cleavage at the GAS surface, potentially by interfering with lysine-dependent interactions involving the Kringle domain 2 (K2) of plasminogen. Earlier studies showed that the GAS plasminogen-binding M - and M - like proteins interact with K2, that contains a lysine-binding site (21–23).

We also see a tendency of increased plasmin activity and a faster plasminogen activation by streptokinase in the presence of TXA. The inhibition of GAS-induced fibrinolysis occurred despite no measurable reduction in plasmin enzymatic activity in the bacterial supernatant or at the bacterial surface, suggesting that TXA interferes likely by blocking lysine-binding sites in plasminogen. It’s known that inhibition of fibrinolysis by TXA does not occur by reducing plasmin activity, but by binding TXA to plasminogen, thereby preventing its interaction with fibrin (24). Consequently, plasmin, generated in solution, cannot bind to and cleave fibrin, resulting in reduced clot lysis despite higher overall plasmin activity. Moreover, it has been previously described that TXA can cause a paradoxical increase in plasmin generation based on *in vitro* and *in vivo* studies (13, 25, 26), and this tendency was also observed in our study using streptokinase or GAS as activator. The mechanism behind this is the change in the conformation of plasminogen due to TXA binding, from a closed to a more open form, which then enhances its activation by both, the host activators urokinase and tissue plasminogen activator (tPa) (13), and apparently by the bacterial activator streptokinase.

TXA at 10 or 20 mg/l did not kill GAS bacteria, incubated in TH medium or plasma. However, in whole blood, TXA significantly reduced GAS proliferation, pointing toward an immune cell dependent mechanism. Flow cytometric analysis of human blood revealed that *ex vivo* GAS infection reduced CD169^+^ and CD66b^+^ cell populations, a pattern only partially restored by TXA treatment. However, responses varied between individual donors, likely reflecting interindividual differences in innate immune activation. CD169^+^ macrophages do not mediate phagocytosis but are mainly involved in the regulation of the immune system (27). Recent findings temper the significance of CD169^+^ macrophages in controlling dissemination of GAS bacteria (28).

CD66b is associated with activated granulocytes (29), and reduced CD66b expression in response to *ex vivo* GAS infection of neutrophils has been described before (30). Thus, a reduction of CD66b on neutrophils may further explain the resistance to neutrophil-mediated killing and the presence of TXA might reverse this effect.

A similar trend was observed for CD14^+^ monocytes, whose reduction following GAS exposure was also reversed by TXA, albeit not reaching significance, again, likely reflecting interindividual variability among donors. These findings support the notion that TXA might generally stabilize or restore immune cell populations otherwise disrupted by GAS activation of plasminogen.

We further examined the systemic effects of TXA *in vivo* using our murine pneumosepsis model (7). The intranasal TXA dose used in mice (200 µg per mouse, ≈10 mg/kg) was within the same range as the clinically used human inhalation dose (500 mg per dose). However, intranasal TXA administration at those clinically relevant doses did not significantly alter bacterial dissemination, coagulation parameters, or major immune cell distributions in peripheral blood. However, we observed modest reductions in pulmonary cytokines, particularly IL-1β, indicating localized anti-inflammatory effects in the lungs.

Interestingly, while lung inflammation was dampened by TXA, the cardiac immune response was differentially modulated. Based on our prior work, GAS-induced bacteremia is known to provoke a shift in cardiac immune composition toward proinflammatory phenotypes, characterized by increased myeloid and Ly6C^^hi^ monocyte infiltration, expansion of M1-like macrophages, and loss of anti-inflammatory M2-like and yolk-sac-derived macrophages (7). In this study, the selective increase in M1-like macrophages with a simultaneous decrease in M2-like macrophages was significantly enhanced by TXA treatment. These changes suggest that TXA may promote monocyte differentiation toward more proinflammatory macrophages. It has been shown before that intravenous TXA treatment resulted in enhanced immune activation that reduced overall infection rates in patients after cardiac surgery (31).

The apparent paradox between reduced proinflammatory cytokines in the lungs and increased proinflammatory M1-like macrophages in the heart may reflect distinct microenvironmental responses to TXA or differences in tissue-specific immune regulation. TXA has been shown to exert pleiotropic effects on immune cells, partly by directly blocking lysine-dependent interactions between plasmin(ogen) and its receptors on immune cells, such as monocytes and dendritic cells, and by modulating soluble inflammatory mediators (32). Additionally, given the route of administration (intranasal), it is unlikely that TXA reached the heart in sufficient concentrations to act directly, and pharmacokinetic data for this route in mice are not available to confirm systemic exposure. Instead, the observed cardiac immune modulation may be secondary to systemic changes in immune cell priming in the blood.

Taken together, our findings suggest that TXA, beyond its antifibrinolytic properties, modulates the host-pathogen interaction during GAS infection by limiting bacterial exploitation of the fibrinolytic system and altering immune responses in both systemic and tissue-specific contexts. The ability of TXA to preserve clot integrity, restrict GAS escape, and partially restore suppressed immune markers in blood may contribute to the reduced bacterial survival observed *ex vivo*. These insights support further investigation into how antifibrinolytics shape innate immunity and may influence the outcome of invasive infections, especially in clinical scenarios where TXA is already administered. Future imaging studies may clarify how TXA shapes the dynamic interplay between GAS, fibrin, and immune cells during infection.

## Data Availability

The raw data supporting the conclusions of this article will be made available by the authors, without undue reservation.
